# Transcatheter Closure of Atrial Septal Defects in Children, Middle-Aged Adults, and Older Adults: Failure Rates, Early Complications; and Balloon Sizing Effects

**DOI:** 10.1155/2012/584236

**Published:** 2012-06-19

**Authors:** Chodchanok Vijarnsorn, Kritvikrom Durongpisitkul, Prakul Chanthong, Paweena Chungsomprasong, Jarupim Soongswang, Duangmanee Loahaprasitiporn, Apichart Nana

**Affiliations:** ^1^Division of Pediatric Cardiology, Department of Pediatrics, Faculty of Medicine Siriraj Hospital, Mahidol University, Bangkok 10700, Thailand; ^2^Pediatric Cardiology, Stollery Hospital, University of Alberta, Edmonton, AB, Canada T6G 2R3

## Abstract

*Objectives*. To compare the failure ratio and inhospital complications across three age groups of patients and to investigate the effects of balloon sizing on the success and the device diameter. *Methods*. This retrospective review was of 665 patients who had been listed for transcatheter-based closure of ASD between 1999 and 2010. The patients were divided into three age groups: children (<18 years; *n* = 183), adults (18–50 years; *n* = 337), and older adults (>50 years; *n* = 145). Procedural outcomes and early complications were reviewed. Use of balloon sizing was explored for its benefits. *Results*. Overall, failure of closure was 6.6% (*n* = 44). Use of balloon sizing tended to lead to a smaller device/defect ratio that was comparable to procedures without balloon sizing, though it did not predict the success rate (OR 1.4, 95% CI 0.7–2.3). Seven patients reported device embolization (1%). No mortalities were noted. In-hospital complications were 3.4%, with common complications, being vascular complications (1.4%) and cardiac arrhythmia (1.1%). No differences in failure rate or events were found among the three groups. *Conclusion*. Transcather closure of ASD is feasible and safe, regardless of the patient's age. A low rate of early complications was noted. Balloon sizing does not aggravate an oversizing of the device, but does not predict success.

## 1. Introduction

Atrial septal defect (ASD) is a common congenital heart malformation comprising 5.9–10% of all congenital heart diseases (CHD) [1]. The defect may lead to right ventricular volume overload, increased pulmonary pressure, atrial arrhythmia, and paradoxical emboli in later decades. Usually, symptoms worsen with increasing age and according to an ageeffect of the patients' ventricular compliance [2, 3]. The ideal remedy has been to eliminate the left- to right-shunt before it develops its late attritions [3]. Surgical closure has been accepted as the standard option, having nearly 100% efficacy [4]. Nevertheless, transcatheter closure of secundum-type ASD has also been used, after its introduction by King and Mills in 1974 [5]. Currently, the technique has become the preferred option in many institutes, because of its effects on cardiac geometry and performance that are comparable to surgical ASD closure, especially for young adults [6, 7]. Transcatheter closure of ASD should be carefully considered for small children, based on the procedure's failure rate and safety [8, 9], though in older adults, the procedure is considered to have contentious benefits, because of long-standing volume overload and ageing comorbidities [2, 10, 11]. 

Transcatheter closure of ASD, using the Amplatzer septal occluder*™*, was introduced in Thailand in 1999. Early outcomes for 26 Thai patients were initially reported by Durongpisitkul et al. in 2000 [12]. Subsequently, comparisons were made between patients who underwent transcatheter closure or surgical closure of ASD in the same institute [13]. In 2008, intermediate outcomes of 76 adult patients in the country were published by Hengrussamee et al. [14]. The number of patients undergoing the procedure has gradually increased in many institutes in Thailand. As the hospital serves for accreditation and quality, the surveillance of in-hospital complications has been conducted in the institute. In this paper, we summarize the 11-year, accumulative failure rates and complications among three age groups: children, middle-aged adults, and older adults, to test our hypothesis that transcatheter closure of ASD is feasible and safe, regardless of the patient's age. In addition, the balloon sizing technique, that usually accompanies the procedure, was retrospectively evaluated for its usefulness, since some studies have claimed that it tends to increase the size of the defects and devices, which may increase the risk of atrial wall erosion and atrioventricular block [15–17].

The purposes of the study were to (1) compare the failure rates and in-hospital complications among three age groups: children and adolescents (<18 years), adults (18–50 years), and older adults (>50 years) and (2) investigate the effects of balloon sizing on the success rate and the device diameter.

## 2. Material and Methods

### 2.1. Data Sources and Variables

This retrospective, descriptive study was approved by the Siriraj Institutional Review Board and Ethics Committee. A total of 665 patients, who had been listed for catheter-based closure of ASD at Siriraj Hospital between February 1, 1999 and February 28, 2010 were identified from the Siriraj Hospital Interventional Cardiovascular Database. All patients who had been diagnosed with isolated secundum ASD and an adequate rim >5 mm, and who were suitable for transcatheter closure by transthoracic echocardiography (TTE) or transesophageal echocardiography (TEE), were eligible for the study.

The 665 consecutive patients were divided into three groups according to age: children and adolescents (<18 years of age; *n* = 183), young to middle-aged adults (18–50 years of age; *n* = 337), and older adults (>50 years of age; *n* = 145), at the time of device closure. A review of the demographic data included age at first presentation, age on the day of procedure, body weight, height, ASD size by intraprocedural TEE, ASD device size, intraprocedural method to confirm the size of defects (TEE, intracardiac echocardiography (ICE), balloon sizing), Qp : Qs, pulmonary artery pressure (measured by right heart catheterization), fluoroscopy time, and procedural time. Success of implantation was defined as the device being properly placed and deployed without malposition or embolization in the catheterization lab. Following the procedure, all major events were recorded to gather information about in-hospital complications. These included cardiac arrest, device embolization, cardiac tamponade, cardiac arrhythmia, hypotension, bleeding (requiring blood transfusion), vascular complications (femoral arteriovenous fistula, femoral hematoma, and femoral thrombosis), stroke, and residual shunt by the day of discharge.

### 2.2. Measurement and Procedure Protocol

All patients were reviewed for their information from physical examinations, chest radiography (CXR), electrocardiograms (ECG), and echocardiographies, before the procedure. Standard transthoracic echocardiographic studies were used to assess ASD morphology, right ventricular systolic pressure (RVSP), and cardiac function to determine the feasibility for transcatheter-based ASD closure. After being informed and giving their consent, patients were scheduled for transcatheter closure of ASD under general anesthesia. Right heart catheterization was performed for all cases to measure pulmonary pressure. Unfractionated heparin (50 unit/kg/dose) and antibiotics were administered intravenously via an arm approach to all patients after femoral access and prior to the procedure. The maximal dose of unfractionated heparin was 3000 unit. Right-heart catheterization was performed for all cases to measure pulmonary pressure. Standard technical delivery of the device over a guide wire through the defect was performed, as previously described [7, 18].

The largest diameter and rim of the atrial septal defect were confirmed by intraprocedural TEE or ICE. In cases where the ASD size and rim were not well visualized in echocardiography, balloon sizing (AGA Medical Corporation, Golden Valley, MN, USA), as a stop flow technique, was used to measure the stretch diameter of the ASD. The ASD device was delivered by an appropriate delivery system and deployed after confirming a satisfactory device position by TEE or ICE and fluoroscopy. When the procedure took more than 60 minutes, the activated clotting time (ACT) was measured and kept to a level of 200–300 seconds. In cases where the ACT was less than 200 seconds, another injection of 25–50 unit/kg/dose of unfractionated heparin was allowed. Following the procedure, patients routinely received six-months of aspirin therapy. All had repeated physical examinations, ECG, and CXR, to determine echocardiographic parameters at 24 hours. A residual shunt was considered if the color Doppler echocardiography revealed left- to right-shunt across the interatrial septum. Patients, who attended the interventional clinic, were evaluated with physical examinations and echocardiographies at: one month, three months, six months, and one year after discharge, and at regular intervals thereafter, according to guidelines.

The outcomes of interest for the study included failure rate of transcatheter closure of ASD for patients who were on the list, and in-hospital complications of patients who had achieved deployment of the ASD device.

### 2.3. Data Analysis

Patients were categorized into three age groups: children and adolescents (<18 years), young and middle-aged adults (18–50 years), and older adults (>50 years). Baseline characteristics of patients and potential confounders were summarized using descriptive statistics and shown in percentage, median, and range. Failure rates were calculated as the ratio among groups. Continuous variables were compared between groups by a One-Way ANOVA for normal distribution variables, and by the Kruskal-Wallis test and the Mann-Whitney *U* test for variables with nonnormal distributions. Category variables were evaluated by the chi-square test and Fisher's exact test. A univariate analysis was performed for each variable with a *P* value < 0.05 considered to be statistically significant. Statistical analyses were performed with SPSS 10.0 for Windows (SPSS.Inc., Chicago, IL, USA).

## 3. Results

### 3.1. Patients' Characteristics

**Figure 1:**
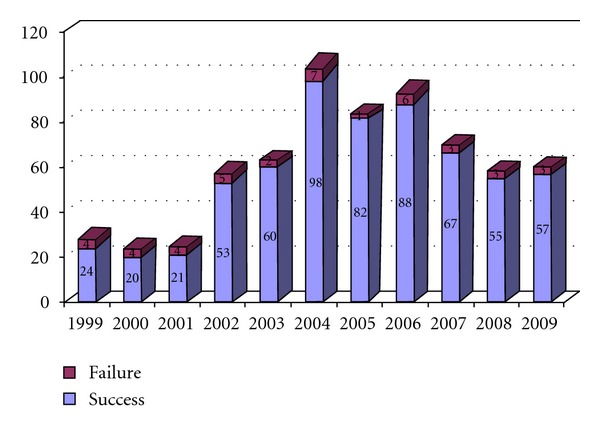
Annual number of cases of transcatheter closure of ASD and failure ratios, Siriraj Hospital (*n* = 665).

During the 11-year study period, a total of 665 patients were scheduled to undergo transcatheter closure of ASD at Siriraj Hospital. Of these, 512 were female and 153 were male (ratio of 77 : 23). The median age at the time of the procedure was 33.5 years (range = 1.2–80.6 years). Of the total number of patients, 27.5%  (*n* = 183) were under 18 years of age, 50.7%  (*n* = 337) were between 18 and 50 years of age, and 21.8%  (*n* = 145) were older than 50 years of age on the day of the operation. Pulmonary artery systolic pressures ranged from 19 to 100 mm Hg, with the lower values being found in pediatric patients. ASD diameter and ASD device ratio in this study was 1.17. The median Qp : Qs was 3.4. Adult patients with Qp : Qs less than 1.5 were indicated to have closure of ASD from suspicion of paradoxical emboli. The median hospital length of hospital stay (LOS) was 1.5 days; however, pediatric patients tended to have longer LOSs, compared to the adult groups. Demographics are shown in Table 1. The number of annually scheduled patients (between February 1999 and February 2010) are shown in Figure 1.

### 3.2. Failure Rates between Age Groups

Of the 665 patients, the failure rate of transcatheter closure of ASD at Siriraj Hospital was 6.6%  (*n* = 44) (Table 2). This included 18 patients (2.7%) who were considered to be unsuitable cases for transcatheter-based closure and failures in 26 patients who attempted to undergo closure of the defect in the cardiac catheterization lab. No statistical difference was found in the failure rates among the three age groups. Specifically, in four patients, intraprocedural TEE revealed holes that were too large (ASD diameter >40 mm) to be treated by transcatheter device closure. In 14 of 18 patients, other abnormalities were present that required surgical repair, such as coronary artery disease and partial anomalous pulmonary venous return. For the 26 patients, devices failed to be placed properly at the defects. Of these, six patients experienced device migration immediately after deployment. For two patients, the device was successfully retrieved percutaneously, and for the other four patients, surgical retrieval and surgical closure of ASD was performed. The other 20 patients experienced difficulties in having the device placed, after attempts were made to either use additional assisting techniques or changing the device to one having a larger diameter. These cases were scheduled for surgical ASD repair, as elective cases.

**Figure 2:**
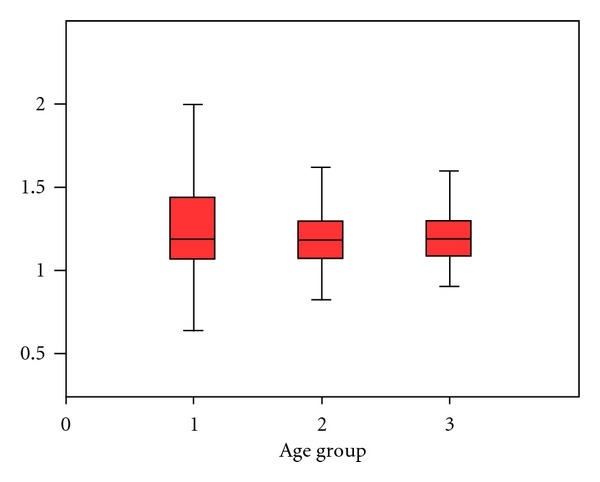
Device/diameter ratio for ASD in successful procedures among the age groups (*n* = 621), age group 1 = children and adolescents (<18 years of age; *n* = 169), age group 2 = young to middle-aged adults (18–50 years of age; *n* = 315), and group age 3 = older adults (>50 years; *n* = 137).

The mean device/diameter ratio for ASD for the 621 patients with successful device implantations was 1.25 ± 0.33 (median = 1.17).  Figure 2 shows the device/diameter ratio for defects among the three age groups. The ratio in the pediatric and adolescent group was 1.30 ± 0.33, which was larger than that in the adult group (18–50 years) (1.22  ±  0.29) and the older adult group (>50 years) (1.25 ± 0.33). For all three groups, the differences were not statistically significant (*P*  value = 0.08). The Amplatzer septal occluder*™* was used mostly (597 patients; 96.3%) in the institute. Two others commercial types of devices the Cocoon septal occluder and the Figulla septal occluder from Occlutech, were used in some selected patients (21 (3.3%) and 3 (0.4%), resp.). 

### 3.3. Impact of Technique with and without Using Balloon Sizing

**Figure 3:**
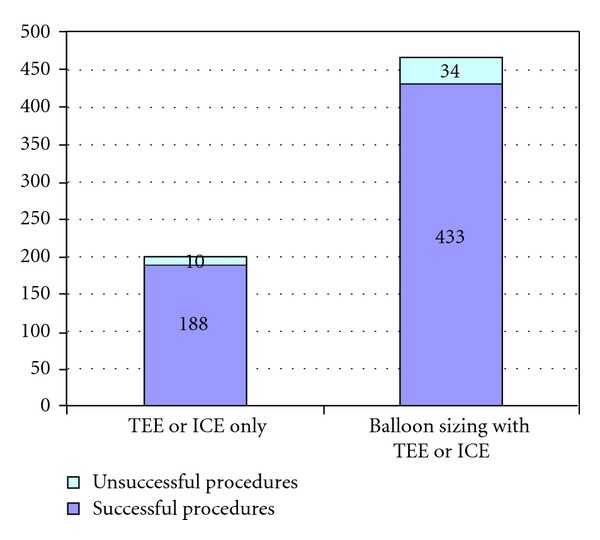
Failure ratios for patients who did, or did not, undergo balloon sizing (*n* = 665). *P* value = 0.29, 95% CI of odd ratio = 0.7–3.5.

**Figure 4:**
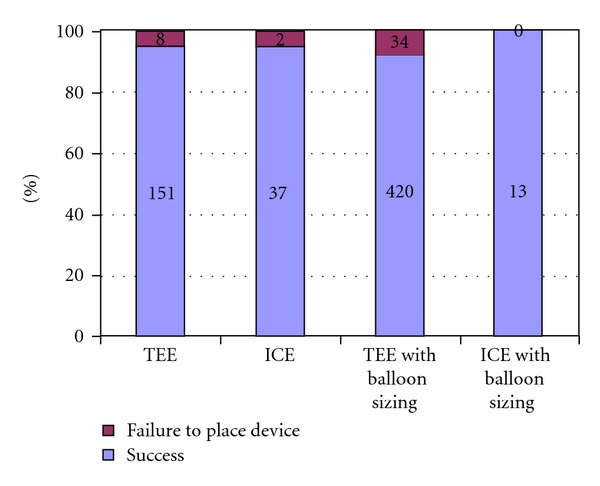
Failure ratio of transcatheter closure of ASD with TEE, ICE, and balloon sizing with TEE and balloon sizing with ICE (*n* = 665). *P* value = 0.52.

In considering the techniques for measuring the defects and for choosing the appropriate device size, we used either balloon sizing or imaging studies on their own. Imaging was obtained by TEE or ICE. The different failure rates, for using balloon sizing and the imaging study on its own, are shown in Figure 3. Of the total number of patients, 70%  (*n* = 467) underwent balloon sizing to measure the defect. Of this number, 34 (7.2%) experienced an unsuccessful placement of the device. Also, 10 (5%) of the 198 patients, who did not undergo balloon sizing, did not have their ASDs closed by the devices. No statistical difference was found in the success rates among those who underwent balloon sizing or imaging studies on their own (OR = 1.4, 95% CI 0.7–2.3, *P*  value = 0.29). Basically, patients who underwent the balloon sizing had larger original defects than the patients who underwent imaging studies only. As a result, greater sizes of ASD devices were required in the prior group (Table 3). Interestingly, when comparing the device and defect ratios, patients who underwent the balloon sizing had smaller ratios, compared to the patients who did not undergo balloon sizing (1.22 ± 0.33 versus 1.31 ± 0.31, resp.).  Figure 4 shows the failure ratios in the modalities using TEE, balloon sizing with TEE and ICE, or balloon sizing with ICE. The pairwise comparisons of using balloon sizing and not using balloon sizing (with TEE) (Table 4) reveal a similar overall trend as in Table 3, while imaging with ICE (Table 5) did not demonstrate any statistical differences, possibly because of the small number of participants.

### 3.4. Early Complications

No in-hospital deaths occurred among the patients who underwent transcatheter-based closure of ASD. Of the 621 patients, 21 (3.4%) had adverse events reported in a recovery ward (Table 6). One pediatric patient, with an initial 11 mm diameter ASD, was reported to have device embolization in the left atrium at six hours after deployment of the Amplatzer septal occluder (14 mm). In the recovery ward, she developed symptoms of nausea, vomiting, and chest discomfort, with occasional premature ventricular beats. A percutaneous retrieval with a gooseneck snare was successfully performed via a femoral vein sheath. TEE, after retrieval, revealed a larger defect (22 mm), according to the avulsion of septum. A second device (24 mm) was then successfully implanted at the defect. TTE confirmed its satisfactory position without a residual shunt at 24 hours, 2 weeks, 1 month, 3 months, 6 months, and 1 year following the procedure. In one older adult patient, a massive pericardial effusion occurred at one hour following the procedure, which was alleviated by a percutaneous pericardiocentesis in the catheterization lab. The pericardial effusion may have been the result of a guide wire perforation. Serious cardiac arrhythmia was observed in seven (1.1%) patients, and one case of atrial fibrillation with fast ventricular response was found in an older adult patient who had been previously diagnosed with paroxysmal atrial fibrillation (AF). The patient's rhythm was converted to a normal sinus rhythm by electrical cardioversion and she continued with medical treatment. Six patients were reported to have transient AV block or a junctional rhythm rate of under 60/min, which were spontaneously resolved without treatment, to stable hemodynamics. Vascular complications were experienced by nine (1.4%) patients. Three of the patients had vascular bruit at groin due to arteriovenous fistula, which required urgent surgical vascular repair. One case of a 42-year-old man with postprocedural hemorrhagic stroke was detected with coincidental cerebral aneurysm after the procedure. No history was reported of abnormal bleeding, chronic headache, systemic arterial hypertension, or cardiac arrhythmia in his previous health record. Routine unfractionated heparin (3000 units) was given before the procedure. A 24 mm, single secundum ASD with adequate rims was demonstrated by intraprocedural TEE. No interatrial aneurysm was noted. A 28 mm Amplatzer septal occluder*™* was placed and deployed uneventfully with no residual shunt or thrombus. At 10 hours after the procedure, the patient developed conscious deterioration. The cerebral computerized tomography and angiography revealed intraparenchymal hemorrhage at the right parietal area, extending to intraventricular, causing obstructive hydrocephalus with the presence of a posterior cerebral artery aneurysm. He underwent ventriculostomy and craniotomy for removal of the clot, followed by coil embolization. The patient recovered gradually in two weeks after the procedure. Postprocedure ASA was not given in the patient. His postprocedural transthoracic echocardiography at day two, one month, three months, six months, one year, and three years showed a well-seated device without residual shunt. No incidents of acute pulmonary edema, cardiogenic shock, nickel hypersensitivity or renal failure were found in the study. At 24 hours after the procedure, echocardiography revealed residual left- to right-shunt in 5.3% of the cases. No significant differences were found in any of the early complications among the three groups. Regarding commercial types, no uneventful complications postprocedurally were reported for the Cocoon septal occluder or the Figulla septal occluder, in keeping with the fact that they were only used for a minority of selected cases.

## 4. Discussion

In the surveillance of cases, the failure ratio of ASD device implantation was 6.6% of the intentions to treat. No significant differences were found in the failure ratio among children, middle-aged adults, and older adult groups. The device/defect ratio was 1.25 ± 0.33. The predicted device size appears to be approximately equal to the original diameter of defect plus 4.2 mm. The balloon-sizing technique was used to augment the procedure in 70.1% of the patients, and the balloon-sizing method appears to have no affect on the success rate (OR 1.4, 95% CI 0.7–2.3). Nevertheless, a bias seems to be present so that balloon sizing is performed generally in large complex or marginal septum atrial septal defects, to more precisely measure the diameter of stretching in the defect by the stop-flow technique. Accordingly, larger defects and larger devices tend to be associated with the use of balloon sizing. Also, the ratio between device and original defect size was found to have a converse relationship in the study. Smaller ratios are more often associated with patients who underwent balloon sizing. This suggests that, in this institute, balloon sizing does not lead to the oversizing of devices, which is in contrast to prior retrospective studies [15, 16]. This finding supports the beneficial use of balloon sizing to assist operators in estimating the appropriate size of devices, especially in difficult cases. In any case, operators should avoid overstretching the balloon, following general recommendations [14]. In general, a shift in the population demographics has occurred, with greater numbers of patients in the older adult age groups. This trend seems to be similar to that seen in other international centers [8, 18, 19]. Based on the findings of this study, the failure ratios for the three age groups are not significantly different.

The impact of age and the procedure's success rate has been mentioned in some prior studies, though no studies have provided data that compares three age groups. Rastogi and colleagues [20] determined the factors related to successful ASO implantation in a small population (*n* = 69) and concluded that patients' weight, diameter of defect, device size, aortic rim, and device/defect ratio were considerably related to their composite success, whereas patient age and consultant experience were not important factors. Recently, in a large cohort study of 1,013 ASD patients, Butera and colleagues [18] suggested that the success of transcatheter closure of ASD depended on accurate measurement of the defect morphology and appropriate device selection. In the study, the authors determined the success rates in two populations: children and adults (>16-years) to be similar (96.1% and 97.8%, resp.). In a comparative study of 236 adult patients with ASD, Humenberger et al. [19] reported on the safety and benefits from a regression of right ventricular size and pulmonary pressure, even among patients of advanced age.

This surveillance found that procedure-related in-hospital complications occurred in 3.4% of cases. No cases of mortality or cardiac perforation were found. The most common events were vascular complications and cardiac arrhythmia, which occurred similarly across all age groups. Serious life-threatening events, such as cardiac tamponade, possibly due to guide-wire perforation, occurred in one older adult patient and was resolved after pericardiocentesis. A hemorrhagic stroke was reported in one patient who had been unaware that he had a coincidental cerebral aneurysm prior to a procedure. The use of unfractionated heparin, which is applied routinely, may have lead to the complication in this case. Regarding device embolization, seven cases were reported; six occurred immediately in the cardiac catheterization lab, and one was detected at six hours after the procedure. Surgical management of the complications was needed in four patients. Overall, in our institute, surgical rescue for serious complications of transcatheter closure of ASD occurred in 0.7% of the cases. Adverse events and complications were not found to differ across the three age groups. In terms of residual shunt, the percentage of residual defect was 5.3% in the 621 patients at 24 hour after the procedure. The ongoing, longitudinal preliminary findings for 353 patients show a reduction to 0.5% at 1 year after the procedure.

Device embolization has long been reported as one of the serious intraprocedural and postprocedural concerns. Prior studies [4, 21, 22] have claimed a prevalence of this complication, from 0.1–3.5% at various centers and with different device types. Albeit the majority of occurrences occurred within 24 hours, some cases of late embolization were reported from 20 days to 2 months. In general, it is difficult to clearly define the cause of embolization, since it may occur even after the operator has checked the device stability. In many studies, the defect was reported to appear after larger postdevice migrations. Accordingly, one hypothesis may be that the chosen device was of an inappropriate size for the defect. After device deployment, the defect may become torn and may have a size which is larger than the original measurement. For overall complications, several reports indicate the early and intermediate adverse events after the procedure. In-hospital complications were reported in 8.65% of cases in a large review by Chessa et al. [22] with the most common early complication being due to device malposition/embolization. Major complications, such as wall erosion/cardiac perforation that were reported in 0.05–0.3% of cases [21, 22], were not found in this study. Long-term results, representing meaningful outcomes of the procedure, have been reported in terms of late complications, clinical improvement, cardiac remodeling, and electromechanical changes [6, 19, 23–25] The issue of age-impact outcomes continues to be an ongoing debate and requires further analysis. 

In summary, the number of accumulative failures and in-hospital complications was found to be relatively small in this study, which reflects the safety of the procedures, even among pediatric or older adult patients. Balloon sizing, used for some patients (70%), does not increase the success or affect the device oversizing. The most common early complications are vascular complications and cardiac arrhythmia. Surgical rescue for serious device complications was reported for 0.7% of cases. These findings support the use of ASD device closure as a procedure with high efficacy and safety, regardless of the patients' age group.

## 5. Limitations

(1) In general, selection bias is inevitable in retrospective studies. Some informative clinical parameters, such as functional capacities, ECG, PR interval, QT dispersion, complete echocardiographic measurement of ventricular function and deformation, and morphological aspect of ASD, were not available. In this study, we gathered as many values as possible, often relying on the intention to treat.

(2) Meaningful outcomes should be based on longitudinal followup, to consider both functional capacities and cardiac adaptation after treatment. In this study, the surveillance of in-hospital outcomes indicates that early complications and failure rates should be considered with caution. With aggregate databases over longer periods and with more inter-institutional cases, overall outcomes and the nation-wide improvements in quality of care for patients may be determined.

## 6. Conclusions

The transcather closure of ASD procedure at Siriraj Hospital has high efficacy and safety, regardless of patient's age at the time of the procedure. From this study, the balloon sizing technique does not affect the success rate or cause oversizing of the device. A low rate of early complications was also noted. More long-term investigations may provide additional valuable information.

## Figures and Tables

**Table 1:** Patients' baseline characteristics (*n* = 665).

	Overall (*n* = 665)	Children and adolescents (<18 years) (*n* = 183)	Young and middle-aged adults (18–50 years) (*n* = 337)	Older adults (>50 years) (*n* = 145)
Age at presentation (years)	32.7 (0.6–80.5)	8.1 (0.6–18)	34.6 (13–49)	59.1 (37–80)
Waiting period prior to procedure (months)	3.14 (0.1–53)	3.6 (0.5–53)	3.1 (0.5–44)	2.6 (0.5–19)
Age at time of procedure (years)	33.5 (1.2–80.6)	8.8 (1.2–18)	35.5 (18.1–49.9)	59.8 (50–80.6)
Weight (kg)	47.9 (9.4–106)	28.3 (9.4–71)	54.9 (28.6–106.5)	56.2 (35.0–86.7)
Height (cm)	148.9 (65–187)	125.3 (75–178)	159.1 (108–186)	156.1 (140–187)
ASD diameter by TEE (mm)	22.1 ± 7.3	17.8 ± 0.7	23.9 ± 6.5	23.4 ± 6.4
Device size (mm)	26.2 ± 7.5 27 (6–40)	20.8 ± 7.9 20 (6–40)	28.2 ± 6.2 28 (10–40)	28.2 ± 6.6 28 (6–40)
Intraprocedural method for confirming the size of defect				
Exclusive TEE	159 (23.9%)	52 (28.4%)	68 (20.2%)	35 (26.9%)
Exclusive ICE	39 (5.8%)	8 (4.3%)	25 (7.4%)	6 (4.1%)
Balloon sizing and TEE	454 (68.2%)	122 (66.6%)	235 (69.7%)	97 (66.9%)
Balloon sizing and ICE	13 (1.9%)	1 (0.5%)	9 (2.7%)	3 (2.1%)
Pulmonary artery systolic pressure (mmHg)	38.6 (19–100)	34.5 (30–57)	40.7 (19.3–100)	43.8 (19–90)
Mean pulmonary artery pressure (mmHg)	21.2 (7–48)	18.6 (11–30)	21.9 (11–48)	23.4 (12–45)
Qp : Qs	3.4 (1.1–26)	2.7 (1.5–16)	3.7 (1.1-20)	3.5 (1.1–26)
Procedural time (min)	56.7 (20–180)	61.7 (20–135)	54.4 (20–180)	56.3 (20–150)
Fluoroscopy time (min)	12.7 (5–100)	14.2 (3–100)	11.7 (3–44)	13.1 (3–70)
Hospital LOS (days)	1.56 (1–88)	2.4 (1–88)	1.3 (1–30)	1.2 (1-2)

Values are expressed as mean ± SD, median (range) and *n* (% within group).

**Table 2:** Success and failure of transcatheter closure of ASD at the end of procedure (*n* = 665).

	Overall (*n* = 665)	Children and adolescents (<18 years) (*n* = 183)	Young and middle-aged adults (18–50 years) (*n* = 337)	Adults (>50 years) (*n* = 145)	*P* value
Device successfully implanted at the end of procedure	621 (93.4%)	169 (92.3%)	315 (93.5%)	137 (94.5%)	NS (0.83)
Unfeasible defects (by TEE), attempt to use ASD device abandoned	18 (2.7%)	3 (1.7%)	11 (3.3%)	4 (2.8%)	NS
Failure of device placement	26 (3.9%)	11 (6.1%)	11 (3.3%)	4 (2.8%)	NS
Device embolization immediately postdeployment	6 (0.9%)	2 (0.5%)	3 (0.8%)	1 (0.6%)	

Values are expressed as *n* (% within group).

^∗^Statistical significance at *P* value <0.05.

**Table 3:** Overall comparison of ASD diameter, device size, device/diameter of defect ratio, procedural time, and fluoroscopy time in successful cases of patients who had, or had not, used balloon sizing (*n* = 621).

	TEE or ICE only (*n* = 188)	Balloon sizing with TEE or ICE (*n* = 433)	*P* value	95% CI of the difference
ASD diameter (mm)	20.5 ± 7.9	22.8 ± 6.9	<0.001^∗^	1.1–3.5
Device size (mm)	25.2 ± 8.0	26.7 ± 7.3	0.03^∗^	0.15–2.7
Device/ASD diameter ratio	1.31 ± 0.31	1.22 ± 0.33	<0.001^∗^	0.03–0.14
Procedural time (min)	54.7 ± 16.6	57.2 ± 23.6	0.14	−6.6–1.2
Fluoroscopy time (min)	10.4 ± 5.7	13.8 ± 8.9	<0.001	2–4.7

Values are expressed as mean ± SD.

Statistical significance at *P* value <0.05.

**Table 4:** Comparison of ASD diameters, device sizes, device/diameter ratio, and procedural times in successful cases of patients who had TEE only or TEE with balloon sizing.

	TEE only (*n* = 151)	TEE with balloon sizing (*n* = 420)	*P* value	95% CI of the difference
ASD diameter (mm)	20.1 ± 8.2	22.9 ± 6.9	<0.001^∗^	−4.0–−1.4
Device size (mm)	24.7 ± 8.4	26.7 ± 6.3	0.004^∗^	−3.41–−0.66
Device/ASD diameter ratio	1.31 ± 0.32	1.22 ± 0.34	0.005^∗^	0.02–0.15
Procedural time (min)	55.8 ± 15.7	57.8 ± 23.7	0.3	−5.9–1.9
Fluoroscopy time (min)	10.2 ± 6.1	13.9 ± 9.0	<0.001^∗^	−5.2–−2.2

Values are expressed by mean ± SD.

^∗^Statistical significance at *P* value <0.05.

**Table 5:** Comparison of ASD diameters, device sizes, device ratios, and procedural times in successful cases of patients who had ICE only or ICE with balloon sizing.

	ICE only (*N* = 37)	ICE with balloon sizing (*n* = 13)	*P* value	95% CI of the difference
ASD diameter (mm)	21.6 ± 7.02	19.4 ± 5.8	0.33	−2.1–6.5
Device size (mm)	27.6 ± 6.0	24.9 ± 6.0	0.12	−1.3–6.8
Device : ASD diameter ratio	1.32 ± 0.23	1.21 ± 0.12	0.23	−0.07–0.29
Procedural time (min)	49.9 ± 21.3	53.8 ± 20.6	0.56	−17.5–9.7
Fluoroscopy time (min)	11.4 ± 4.0	10.5 ± 3.8	0.50	−1.7–3.4

Values are expressed as mean ± SD.

^∗^Statistical significance at *P* value <0.05.

**Table 6:** In-hospital complications following transcatheter closure of ASD (*n* = 621).

	Overall (*n* = 621)	Children and adolescents (<18 years) (*n* = 169)	Young and middle-aged adults (18–50-years) (*n* = 315)	Older adults (>50-years) (*n* = 137)	*P* value between group
Cardiac arrest	0	0	0	0	NS
Device embolization	1 (0.1%)	1 (0.5%)	0	0	NS (0.26)
Cardiac tamponade	1 (0.1%)	0	0	1 (0.7%)	NS (0.17)
Tachyarrhythmia ventricular rate > 160/min	1 (0.1%)	0	0	1 (0.7%)	NS (0.17)
Bradyarrhythmia ventricular rate < 60/min	6 (0.9%)	2 (1.1%)	3 (0.9%)	1 (0.7%)	NS (0.92)
Hypotension	0	0	0	0	NS
Bleeding and requiring blood transfusion	1 (0.1%)	1 (0.5%)	0	0	NS (0.26)
Vascular complication	9 (1.4%)	4 (2.3%)	3 (0.9%)	2 (1.4%)	NS (0.86)
(i) Femoral arteriovenous fistula	3 (0.3%)	0	2 (0.6%)	1 (0.7%)	
(ii) Femoral hematoma	4 (0.6%)	2 (1.1%)	1 (0.3%)	1 (0.7%)	
(iii) Femoral thrombosis (resolved by heparin infusion)	2 (0.4%)	2 (1.1%)	0	0	
Cerebrovascular accident	1 (0.1%)	0	1 (0.3%)	0	NS (0.61)
Renal failure	0	0	0	0	NS
Nickel allergic reaction	0	0	0	0	NS
Residual shunt by TTE postprocedure 24 hours	33 (5.3%)	10 (5.3%)	14 (4.4%)	10 (0.7%)	NS (0.59)

Values are expressed as median (range) and *n* (% within group)

^∗^Statistical significance at *P* value <0.05.

## Abbreviations

AF: Atrial fibrillation
ASD: Atrial septal defect
CHD: Congenital heart disease
CXR: Chest radiography
ECG: Electrocardiogram
ICE: Intracardiac echocardiography
LOS: Length of hospital stay
RVSP: Right ventricular systolic pressure
TEE: Transesophageal echocardiography
TTE: Transthoracic echocardiography.
